# Machine Learning for detection of viral sequences in human metagenomic datasets

**DOI:** 10.1186/s12859-018-2340-x

**Published:** 2018-09-24

**Authors:** Zurab Bzhalava, Ardi Tampuu, Piotr Bała, Raul Vicente, Joakim Dillner

**Affiliations:** 1 Dept. of Laboratory Medicine, Karolinska Institutet, F46, Karolinska University Hospital Huddinge, Stockholm, Sweden; 2 Institute of Computer Science, University of Tartu, Tartu, Estonia; 3 Interdisciplinary Centre for Mathematical and Computational Modelling, University of Warsaw, Warsaw, Poland

**Keywords:** Machine learning, Metagenomic sequencing, Human samples, Viral genomes

## Abstract

**Background:**

Detection of highly divergent or yet unknown viruses from metagenomics sequencing datasets is a major bioinformatics challenge. When human samples are sequenced, a large proportion of assembled contigs are classified as “unknown”, as conventional methods find no similarity to known sequences. We wished to explore whether machine learning algorithms using Relative Synonymous Codon Usage frequency (RSCU) could improve the detection of viral sequences in metagenomic sequencing data.

**Results:**

We trained Random Forest and Artificial Neural Network using metagenomic sequences taxonomically classified into virus and non-virus classes. The algorithms achieved accuracies well beyond chance level, with area under ROC curve 0.79. Two codons (TCG and CGC) were found to have a particularly strong discriminative capacity.

**Conclusion:**

RSCU-based machine learning techniques applied to metagenomic sequencing data can help identify a large number of putative viral sequences and provide an addition to conventional methods for taxonomic classification.

**Electronic supplementary material:**

The online version of this article (10.1186/s12859-018-2340-x) contains supplementary material, which is available to authorized users.

## Background

A large number of different viruses are present in biospecimens from humans [1, 2]. The proportion of viral sequences and its composition seem to change in diseased individuals [3, 4]. As many novel viruses are continuously discovered, it is possible that many human viruses are yet to be detected [5–10]. Next Generation Sequencing (NGS) technologies are used to directly examine the DNA present in clinical samples, without the requirement of prior information about sequences that may be present [11]. Metagenomics refers to the complete sequencing of all microbiological genomes in a biospecimen and viral metagenomics is routinely used for virus detection and discovery of new viruses [5, 9, 10, 12–17]. In order to detect potential viral sequences in metagenomic datasets, conventional alignment-based classification is performed by BLAST, which compares sequences to known genomes and calculates how much similarity they share. A downside of the method is that public databases for virus-related genomes are incomplete. A large number of sequences are labeled as “unknown” since many of them have only very distant or no homologs in public databases [5, 7].

The HMMER3 algorithm implements Hidden Markov Models with a reference set of sequences encoding viral proteins (“vFams”) [18]. This method appears to be more effective in detecting distant homologs in metagenomic datasets [19]. However, it is also dependent on a reference database such as “vFams”, which like any other public database is incomplete. Predictive models (for example built via machine learning algorithms), on the other hand, use a training database only to learn what the relevant features and criteria for classification are, and can then be applied to any new data point. In particular, we propose that machine learning methods as presented in our work can act as a recommendation system to sort and prioritize the sequences marked as “unknown” by existing methods for further research.

In this work, we wished to investigate whether machine learning using the relative synonymous codon usage (RSCU) in the sequences could be used to predict the presence of human virus sequences in metagenomic NGS data. In the genetic code, some amino acids are encoded by several, synonymous codons. Usage of these codons is not random and differs among species. This phenomenon is called Codon Usage Bias. Several viral families (in particular the herpesvirus, lentivirus, papillomavirus, polyomavirus, adenovirus, and parvovirus) are known to encode structural proteins that display heavily skewed codon usage compared to the host cell [20, 21].

In order to test whether codon usage could predict viral nature of a sequence we used genes extracted from NCBI GenBank to build a virus/non-virus classifier. A cross-validation approach using Random Forests achieved almost perfect accuracy on this dataset. However, the models trained on NCBI GenBank data fail to generalize to classifying contigs obtained from metagenomics analysis, with random-like performance. As the main contribution, we trained Random Forests and neural networks on a metagenomic sequencing dataset generated by NGS technologies applied to human biospecimens. As we wished to develop an algorithm to detect presence of human viruses, viruses infecting bacteria (phages) were not included in the training set. We show that models trained using RSCU values from contigs from a set of metagenomics experiments generalizes to other metagenomics experiments. Furthermore, we investigated which codons were more important for the models to classify a sequence as a virus.

## Methods

### Dataset

#### Patients and samples

Next Generation Sequencing (NGS) using the Illumina platform was used to generate the metagenomic sequencing datasets from human samples coming from several different patients groups, as described [6–8, 22–24]. The purpose of all of these studies was to investigate the presence of viral genomes or other microorganisms in human biospecimens from patients who developed diseases or from matched control subjects. Further information of the samples is provided in Additional file 1.

#### Sequencing

Sequences were generated from the MiSeq, NextSeq and HiSeq (Illumina) sequencing platforms, as described by the manufacturer. When multiple human samples were included in the same sequencing run, the sequences were mapped to the originating sample using sequence indices, included in the Illumina adapters.

#### Bioinformatics pipeline

**Fig. 1:**
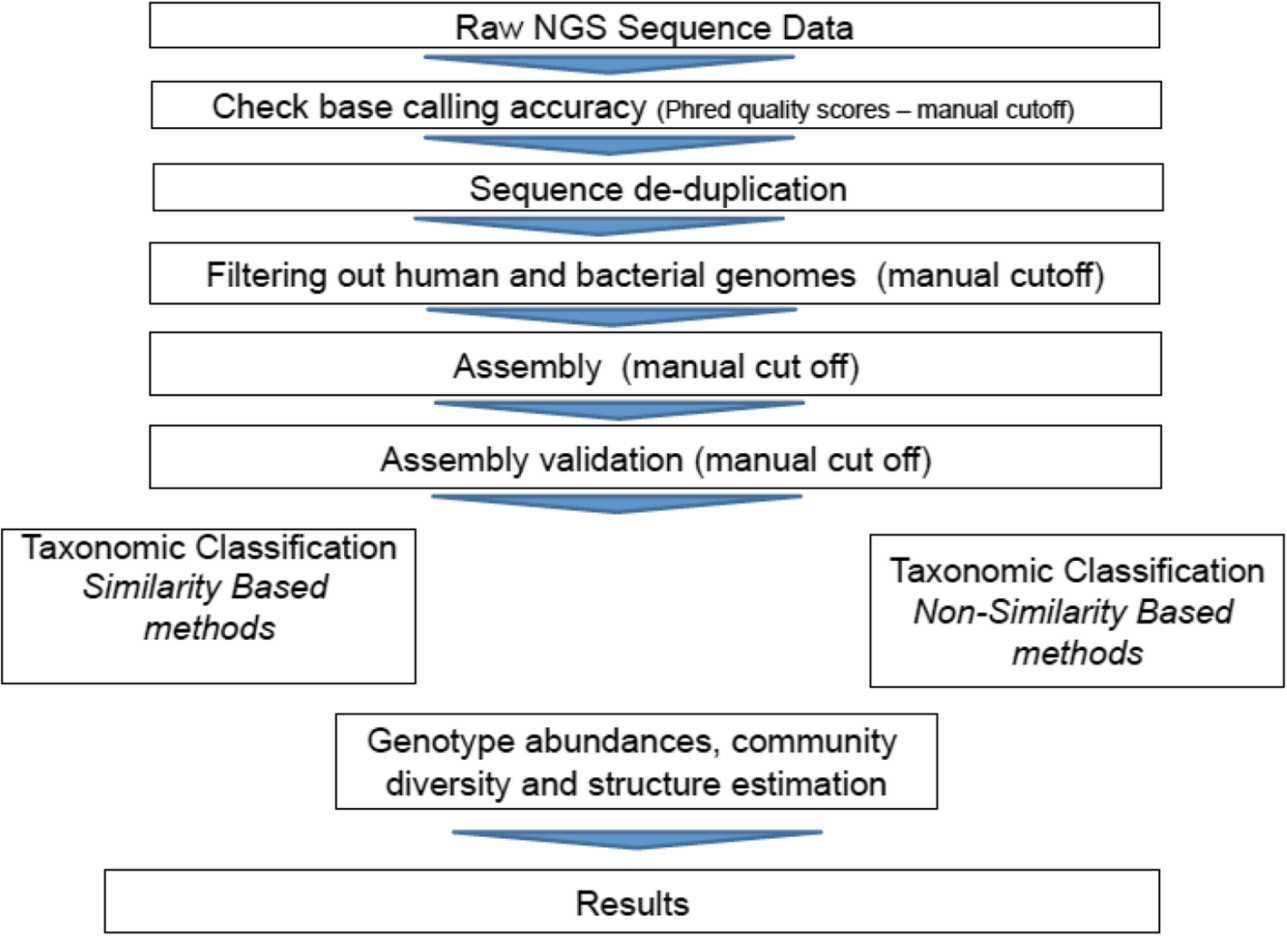
Flow of the bioiformatics pipeline of NGS data for viral metagenomics [25]

Before applying machine learning techniques, all sequencing experiments were analyzed using a benchmarked bioinformatics pipeline, as described [25]. The pipeline starts with quality checking and reads are trimmed according to their Phred quality scores. After this, reads that are highly similar (with 95% identity over 75% of their length) to human, phage, bacterial and vector DNA are removed from further analysis using BWA-MEM [26]. The rest of the reads are normalized and then processed for assembly using the Trinity [27], SOAPdenovo, SOAPdenovo-Trans [28] and IDBA-UD [29] assemblers. we used several assembly algorithms in order to validate results. Then the assembled contigs are subjected to taxonomic classification using alignment-based classifiers such as BLAST and HMMER3. The code of the pipeline is available on GitHub (https://github.com/NIASC/VirusMeta and https://github.com/NGSeq/ViraPipe). Different steps of the pipeline are shown in Fig. 1.

#### Feature extraction and labeling

Sequencing datasets were obtained from 19 different NGS experiments. After the de novo genome assembly, two different algorithms were applied for viral classification. Firstly BLASTn algorithm (reward for nucleotide match =1; penalty of nucleotide mismatch =1; cost to open a gap =0; cost to extend a gap =2; e-value ≤*e*^−4^) was applied with NCBI nucleotide database. We also re-analysed the assembled contigs by PCJ-BLAST [30] with the most recent version of nt database. Number of contigs classified by BLAST into different taxonomy groups is shown in Additional file 2. For the contigs that were classified as unknown we used HMMER3 algorithm. As reference database for this algorithm, we used a database which includes viral profile hidden Markov models (“vFams”) from all the virally annotated proteins in RefSeq (http://derisilab.ucsf.edu/software/vFam) [18]. Both BLAST and HMMER3 results were used for the machine learning. Note that while BLAST classifies sequences in different taxonomic groups, HMMER3 with the “vFam” reference set only identifies viral genomes. All assembled-sequences, classified and labeled by this bioinformatics pipeline were combined to train the machine and deep learning algorithms. This dataset consisted of 3% of viral contigs. Usually, viruses are less than 0.1% in a metagenomic dataset but the removal of highly identical non-viral reads at the initial stage of the analysis relatively increases the proportion of viruses in the dataset.

To extract features from the metagenomic dataset for the machine learning purposes we used Relative Synonymous Codon Usage frequency (RSCU) [31]. Proteins are encoded by 20 different amino acids but there are 64 codons encoding for them. Trinucleotides coding for the same amino acids are called synonymous codons and usage of those is not random: some species prefer one codon over another. For a given contig, we calculate the RCSU value for each codon with the following formula:

1 $$ f_{ij} = \frac{x_{ij}}{\frac{1}{n_{i}}\sum_{j=1}^{n_{i}} x_{ij}}  $$

Where *x*_*ij*_ is the number of occurrences of *j*-th synonymous codon coding for amino acid *i*. *n*_*i*_ is the total number of synonymous codons that encode for amino acid *i*. This ratio can be defined as the observed number of codon occurrences divided by expected usage assuming uniform distribution [31]. Methionine and Tryptophan that have only one corresponding codon (ATG and TGG, respectively) were removed from the analysis since they would not contribute to the study. Furthermore, stop codons were also removed. This gave us a total number of 59 features.

DNA has double strands: forward (5 to 3’) and reverse (3 to 5’). Since in metagenomic sequencing data it is not known from which strand a contig came from, we counted RSCU values for both directions and considered them as two independent samples. Furthermore, because of the fact that RSCU values are counted only in regions of Open Reading Frames (ORFs), assembled-sequences that did not have at least two ORFs in either direction were discarded from the further analysis. In this study, a stretch of codons was considered as an ORF if there was a stretch of at least 120 nucleotides between a start codon and a stop codon.

#### Dataset from NCBI GenBank

The NCBI GenBank sequences were obtained from Codon Usage Database (https://www.kazusa.or.jp/codon/). For this database codon usage (RSCU values) was calculated for complete genes using nucleotide sequences from the Genbank. For the analysis we used approximately 600 thousand proteins from which 14% were viral.

### Machine learning analysis

In the preliminary analysis using Genbank data we first took a 10-fold cross validation approach using Random Forests with different sizes and with/without balancing the class weights. The reported results are the validation performance from the best performing parameter configuration, averaged over the 10 folds. Secondly we trained a Random Forest model on the entire NCBI GenBank dataset and tested its performance on RSCU values from contigs obtained from metagenomics experiments.

In the main body of work we trained models using metagenomics datasets. We used assembled-sequence data from 19 different metagenomic sequencing runs. However, we did not combine all contigs into one big dataset, because contigs from the same run might be highly similar to each other (in terms of hamming distance, for example). We applied cd-hit-est algorithm [32] with sequence identity threshold 0.98 and coverage 0.95 on the entire dataset to remove highly similar assembled-sequences. However, we observed that contigs from the same run are still more likely to be similar than contigs from different runs. We did not want such highly similar sequences to end up in both training and testing data, because it would result in an artificially high accuracy that does not reflect the true ability to generalize to unseen samples. In order to provide an honest estimate of the test accuracy, we took an approach we call *leave-one-experiment-out cross-validation (LOEO)*. With this approach, we trained our machine learning algorithms on data coming from 18 metagenomic sequencing runs and tested them on the remaining 19th run. We repeated this process 19 times and each time data from a different experiment was used as test set. Using this methodology we test algorithms on truly unseen data from an entirely different experiment, which gives us a fairer estimate of the performance of the model compared to a traditional K-fold cross validation.

The leave-one-experiment-out cross-validation approach gave us 19 different models and 19 validation sets of different size and prevalence. In order to combine results from different folds, we used macro and micro averaging approaches [33]. In macro averaging, the number of samples in each validation set is disregarded and the 19 results are simply averaged [33] (we average over datasets). In the micro averaging approach, however, experiments that provide more validation samples have a bigger influence on the results [33] (we average over samples).

In this study, we applied this leave-one-experiment-out approach using two classification algorithms: the main results were obtained using Random Forests, but later we validated the results with Artificial Neural Networks. We have selected to use Random Forest and Neural Networks for several practical and theoretical reasons. Most importantly, the hierarchical structure of the two algorithms allows combining the relatively simple features, such as RSCU values, to form more complex decision boundaries than simple non-linear regression models or SVMs. In addition, the capacity of both algorithms is easily controllable and they are widely used yielding state of the art results in many tasks.

#### Random forest

Random Forest is a collection of a large number of decision trees. Each tree differs from others because it is trained on a different set of training samples and because at each splitting point only a random subset of features are considered [34]. The differences between the trees work together and the average prediction made by the group is more accurate than one individual tree [34]. In this study, we used scikit-learn-0.18.1 implementation in python 2.7 [35]. More thorough description of Random Forests and the hyperparameters we tested is given in Additional file 3.

##### Feature importance in random forest

Each decision tree in a random forest is a collection of simple splitting rules (if-then statements) that use only one feature at a time. At each splitting point only a small subset of features are considered. Among the possible one-feature if-then statements, the rule that maximally reduces Gini impurity is always chosen. Gini impurity is reduced if the two nodes resulting from the rule have a less uniform class distribution than the parent node. How important a feature was in an entire tree can be estimated by summing up the impurity reductions brought about by this feature at all different branching points where it was used [34, 36]. The importance of each feature in each individual tree is calculated and easily accessible to the user in scikit-learn’s RandomForestClassifier [35].

To compare the importance of RSCU values of different codons for our classification task, we averaged the importance of the features across 1000 trees trained on the entire data set of all 19 experiments. When interpreting the mean importance of features, we need to notice that the RSCU values of synonymous codons can be highly correlated - if the value for one synonymous codon is high, the other(s) must be low. If there are only two synonyms, the correlation is almost perfect. Correlated features “compete” for importance - the RSCU value that is used first in a given tree will have the chance to contribute the information shared between the correlated features and is likely to show up as more important [36, 37]. In a different tree the randomness might lead to another feature being selected first and contributing highly. This leads to high variance of feature importance across trees. Despite high variance, we believe the average importance is still interpretable and reveals which codons’ RSCU values are useful more often than others [36], especially when the differences are clearly visible.

#### Artificial neural networks

Artificial Neural Networks is a machine learning algorithm inspired by the structure of the biological networks of neurons in the brain. The simplest type of artificial neural networks, feed-forward neural network (FFN) used in this work, consists of multiple layers of nodes (called “neurons”) and the connections between these nodes [38]. There are no connections between the nodes of the same layer, whereas neighboring layers are all-to-all connected with each other. Each node is characterized by its activation and each connection by its weight. These connection weights can be optimized by providing the network input-output pairs.

To implement neural network models we used version 2.0.5 of Keras library (https://keras.io/) in Python 2.7. The procedure to find useful values for hyperparameters (network size, depth, etc) and a more thorough description of neural network approach is provided in Additional file 3.

## Results

To test whether the relative synonymous codon usage frequency can predict the viral nature of a sequence we firstly trained a model on sequences originating from NCBI GenBank. Secondly, we trained models only on assemebled metagenomics contigs. We show how well these models classify viral sequences and compare results. During this study we used two datasets and in each dataset there was a high class imbalance. Therefore, we used area the under Receiver Operating Characteristic (ROC) curve as our main metric to evaluate the results since it is not dependent on class distribution.

We start with results from a preliminary analysis obtained using the dataset from NCBI Genbank. Thereafter, in the main analysis, we first describe and analyze the results obtained on metagenomics datasets with Random Forest (RF) classifier. Also, we analyze how important role each RSCU value played in the classification. Finally, to validate the results obtained with RF, we demonstrate that a similar classification performance can be achieved with feedforward neural networks.

### GenBank model

**Fig. 2:**
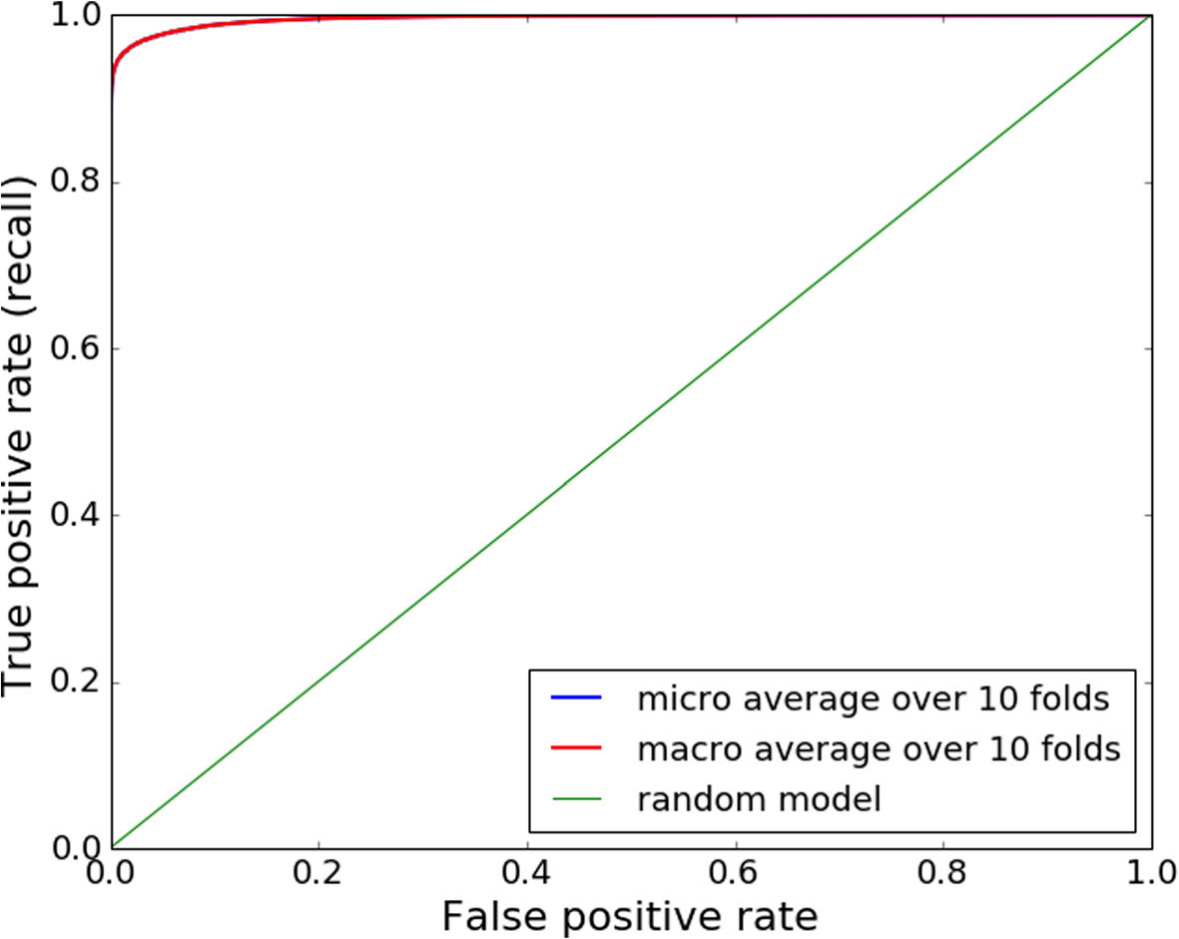
ROC curve of GenBank model when testing on GenBank data. The models trained in 10-fold cross validation, achieved 0.99 area under the ROC curve while classifying RSCU values obtained from genes registered at the GenBank. Notice that in case of equal fold sizes, micro and macro averages are equal

**Fig. 3:**
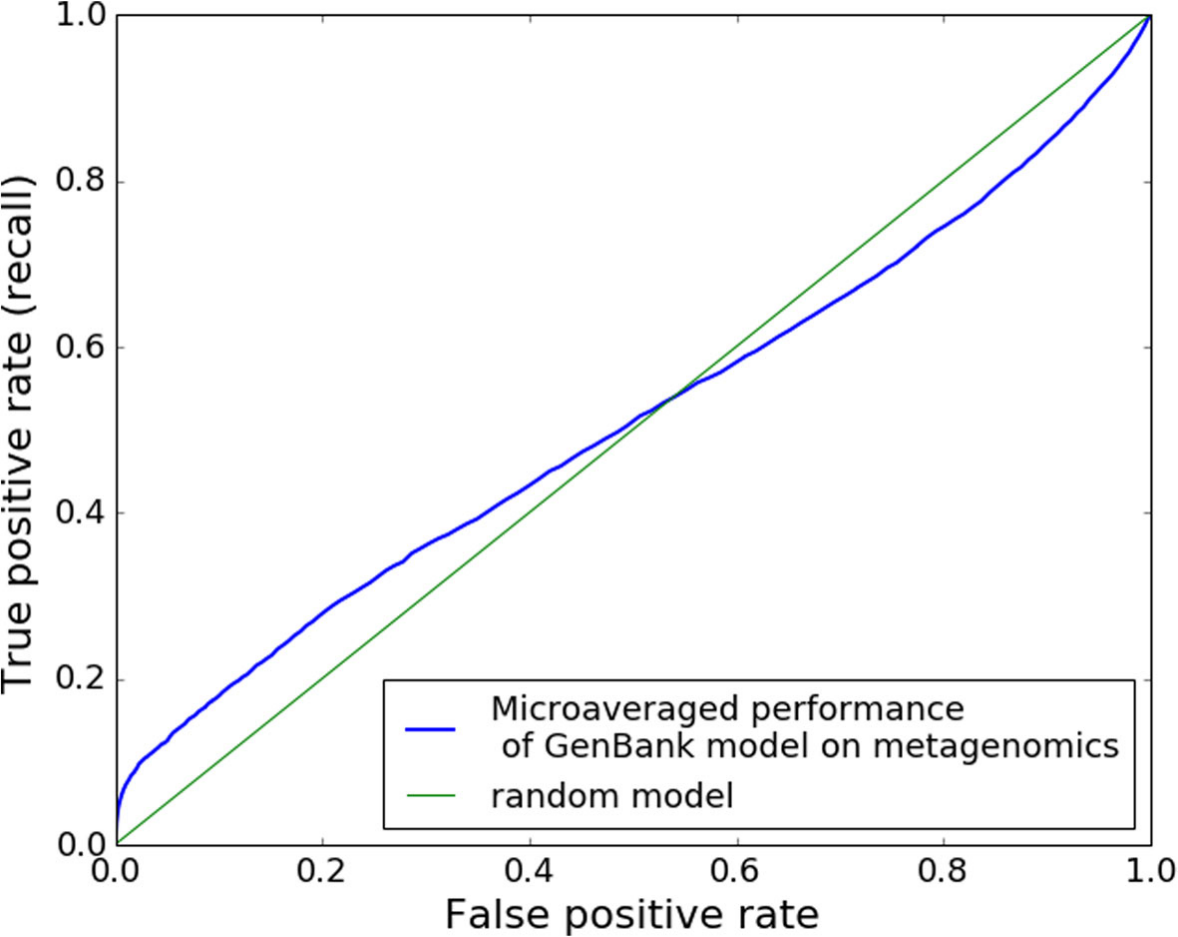
ROC curve of GenBank model when tested on metagenomics data. Area under the ROC curve is 0.51, which means the model trained on GenBank data failed to generalize on metagenomics data. It’s performance is close to random level

The first set of random forest models was trained on RSCU values obtained from genes registered at Genbank. Using k-fold cross validation method (k =10) the approach showed very high accuracy. Figure 2 shows that averaged across all folds the random forests achieve 0.99 area under the ROC curve, meaning that classifying genes based on their RSCU values can be done almost perfectly. As a next step we applied a model trained on GenBank data on metagenomics data in order to see how well it would generalize on this type of noisy dataset. As Fig. 3 shows the model clearly failed to classify the assembled metagenomics contigs. The area under the ROC curve was 0.51. Despite the fact that the same model performed very well on a dataset obtained from Genbank its accuracy on metagenomics was very close to a random classifier.

### Metagenomics model

As our primary goal was to detect viral sequences in metagenomics and given the fact that Genbank model could not classify assembled contigs, we trained the next model entirely on metagenomics data.

We used Random Forests with different hyperparameter combinations (number or trees, up and down sampling, class weights), applying the leave-one-experiment-out (LOEO) type cross-validation (see “Methods” section). The results with different tested hyperparameters are provided in Additional file 4. The best results were obtained with 5000 trees, balanced class weights and no up or down sampling. In the following we present the results from this model, unless clearly stated otherwise.

**Fig. 4:**
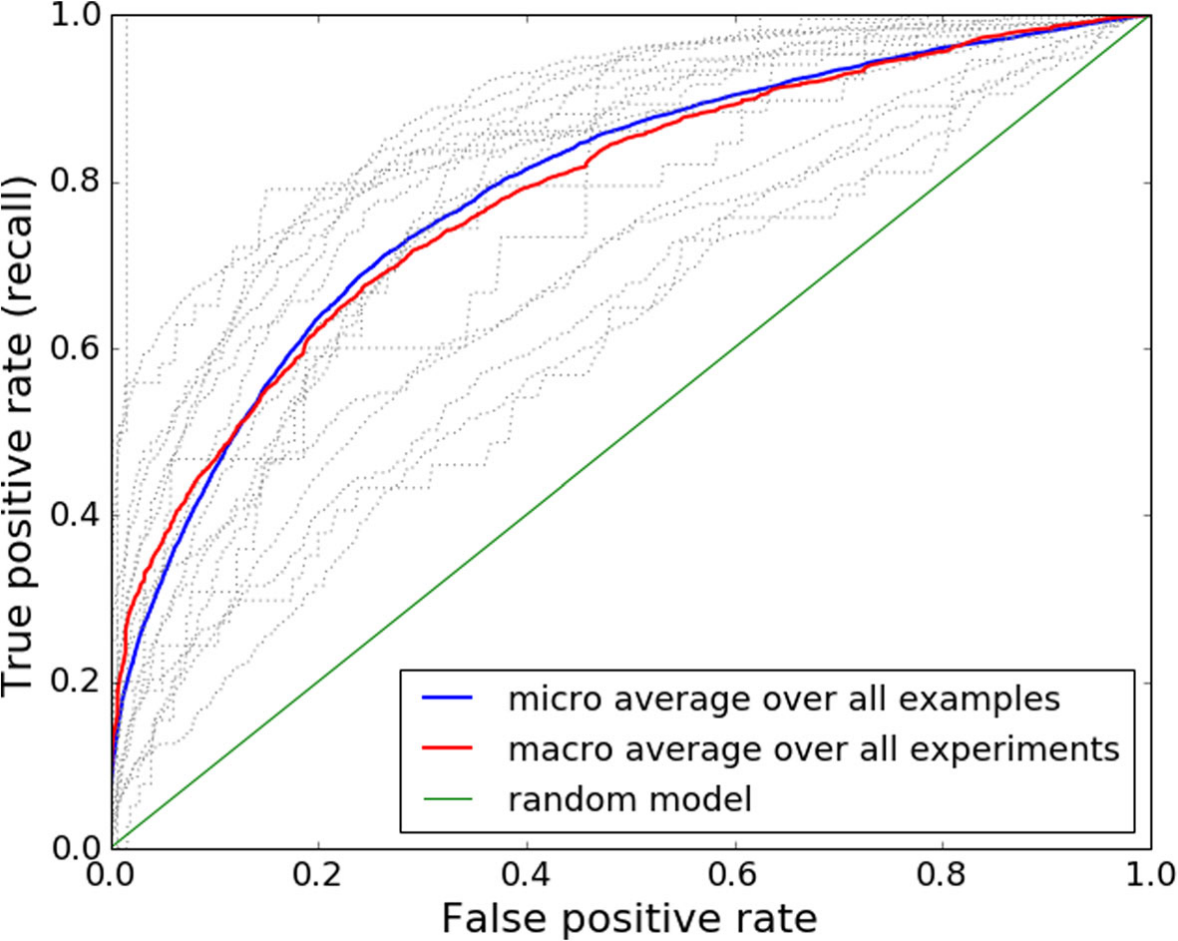
ROC curves of the metagenomics model for each LOEO cross-validation fold and their micro and macro averages. The grey lines depict the ROC curve in each leave-one-experiment-out cross-validation fold. The red line is the macro-averaged curve and blue line the micro-averaged curve. Green line shows the performance of a random model. The averaged results are clearly above the diagonal, meaning the models perform a much better than a random guess

Joining the predictions for all samples and for all experiments in our LOEO cross-validation approach, we can describe the overall performance of the random forests. In Fig. 4 we visualize the ROC curves for each individual validation set (i.e. data from each of the 19 metagenomics experiments, dotted gray lines) as well as the micro-averaged and macro-averaged ROC curves across the 19 validation sets (blue line and red line respectively). The area under the micro-averaged ROC curve is 0.789 meaning the models performed clearly better than a random classifier or the Genbank model. Because our data comes from very different experiments, we also provided the macro-averaged results - statistics from all 19 cross-validation folds are averaged disregarding the number of samples. The area under the macro-averaged ROC curve is 0.785, confirming that the models perform well on data from different experiments. See Additional file 5 for results per validation set (i.e per metagenomics experiment).

#### Precision and recall

The leave-one-experiment-out (LOEO) cross-validation approach yields a probability for each contig being a virus. Across the 19 datasets, we had on average 3% of virus and 97% of non-virus samples. Notice that a naive model that classifies everything as non-virus would have a 97% overall precision and 97% overall recall. Despite rather high overall precision and recall, this model would clearly be useless for separating the classes. With a high class imbalance we needed to describe the precision and recall for both classes separately, instead of overall performance. This way we could gain more insight to the model’s actual ability to detect viral samples. Table 1) summarizes the precision and recall at threshold p(virus) ≥0.5 for the two classes, using micro and macro averaging. We see that the model is clearly doing better than simply classifying all samples as “not virus”.

**Table 1:** Micro- and macro-averaged performance measures across 19 experiments using random forests

Method	Class	Precision	Recall	F1-score
Micro-average	Non virus	0.97	1.00	0.99
Micro-average	Virus	0.92	0.05	0.10
Macro-average	Non virus	0.96	1.0	0.98
Macro-average	Virus	0.53	0.04	0.08

The results presented are from the best model according to area under ROC curve (*ROC*_*micro*_=0.789). This model used 5000 trees, balanced class weights and no down nor upsampling

**Fig. 5:**
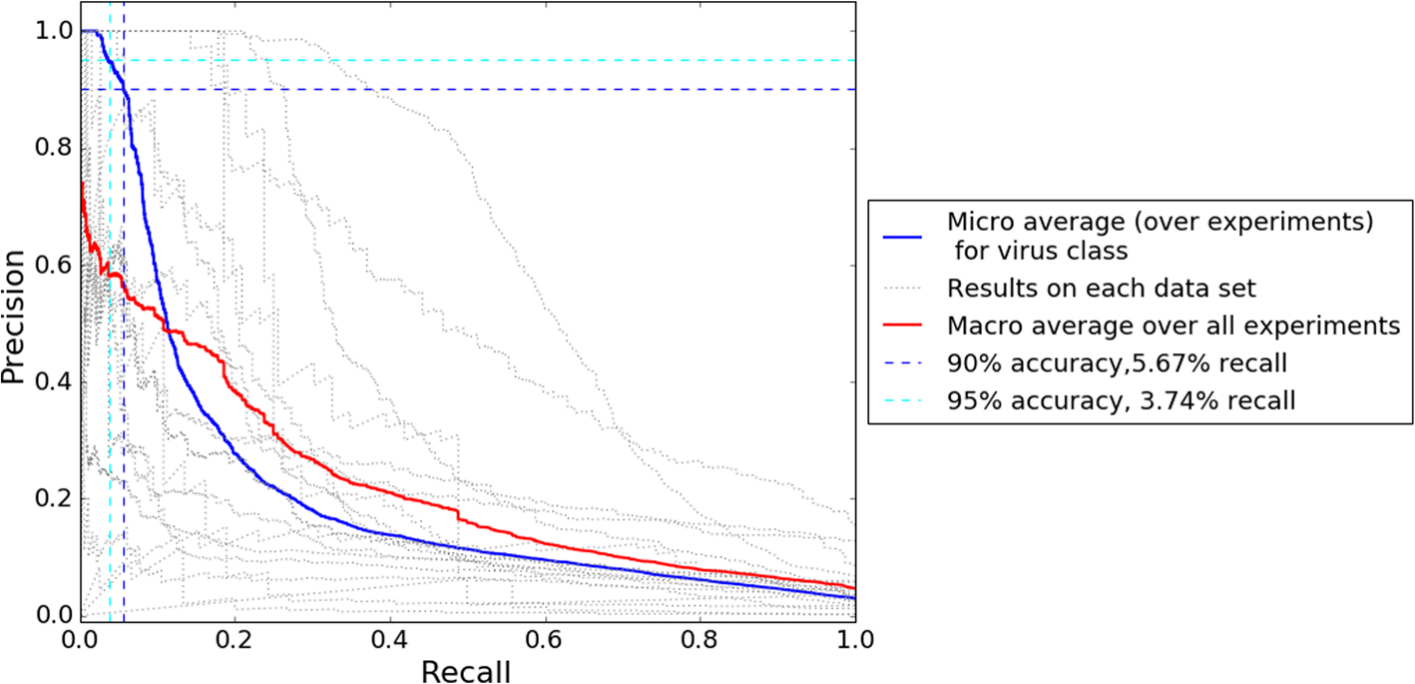
The trade-off between precision and recall for virus vs. non-virus classification when changing the classification threshold. A sample is classified as virus only if p(virus) >threshold. Dotted lines depict the precision and recall for virus class at different thresholds for individual folds (i.e. metagenomics experiments) in LOEO cross validation. Blue line depicts the micro average across the experiments, red line illustrates macro average. The vertical and horizontal dashed lines exemplify what performance can be obtained when boosting precision at the expense of recall. Blue dashed line shows that at 90% accuracy we can obtain 5.67% recall. Cyan dashed line shows that a threshold giving us 95% precision would yield 3.74% recall. The models yielding highest area under ROC curve are used for this graph

In Table 1 we reported the precision and recall if all samples with p(virus) ≥0.5 are classified as virus. This is an intuitive threshold to set - it means that we assign the most probable class to each sample. Notice however, that setting a higher threshold would make the decision stricter and would likely increase the precision at the expense of recall, whereas a lower threshold boosts recall at the expense of precision. Varying the strictness of the classification can be useful depending on the context and purpose of the analysis. If one needs to detect the maximum amount of viruses and is willing to accept many false positives, a low threshold can be useful. Inversely, if false positives are costly to deal with while one can accept letting many viruses pass unnoticed, a high threshold might be useful. This is also the case in metagenomics analysis for finding new viral sequences - setting a threshold yielding high precision might be useful as further biological analysis can be costly. Figure 5 illustrates the trade-off between precision and recall for the virus class using the model with highest area under ROC curve. With this model we can, for example, achieve 75% accuracy at 8.0% recall, 90% accuracy at 5.6% recall and 95% accuracy at 3.7% recall. However, if choosing the model maximizing these recall values instead of maximizing area under ROC curve, we can also achieve 75% accuracy at 10.5% recall, 90% accuracy at 8.6% recall, 95% accuracy at 5.5% recall. See Additional file 4 for results with different hyperparameter values.

### Feature importance and visualization

**Fig. 6:**
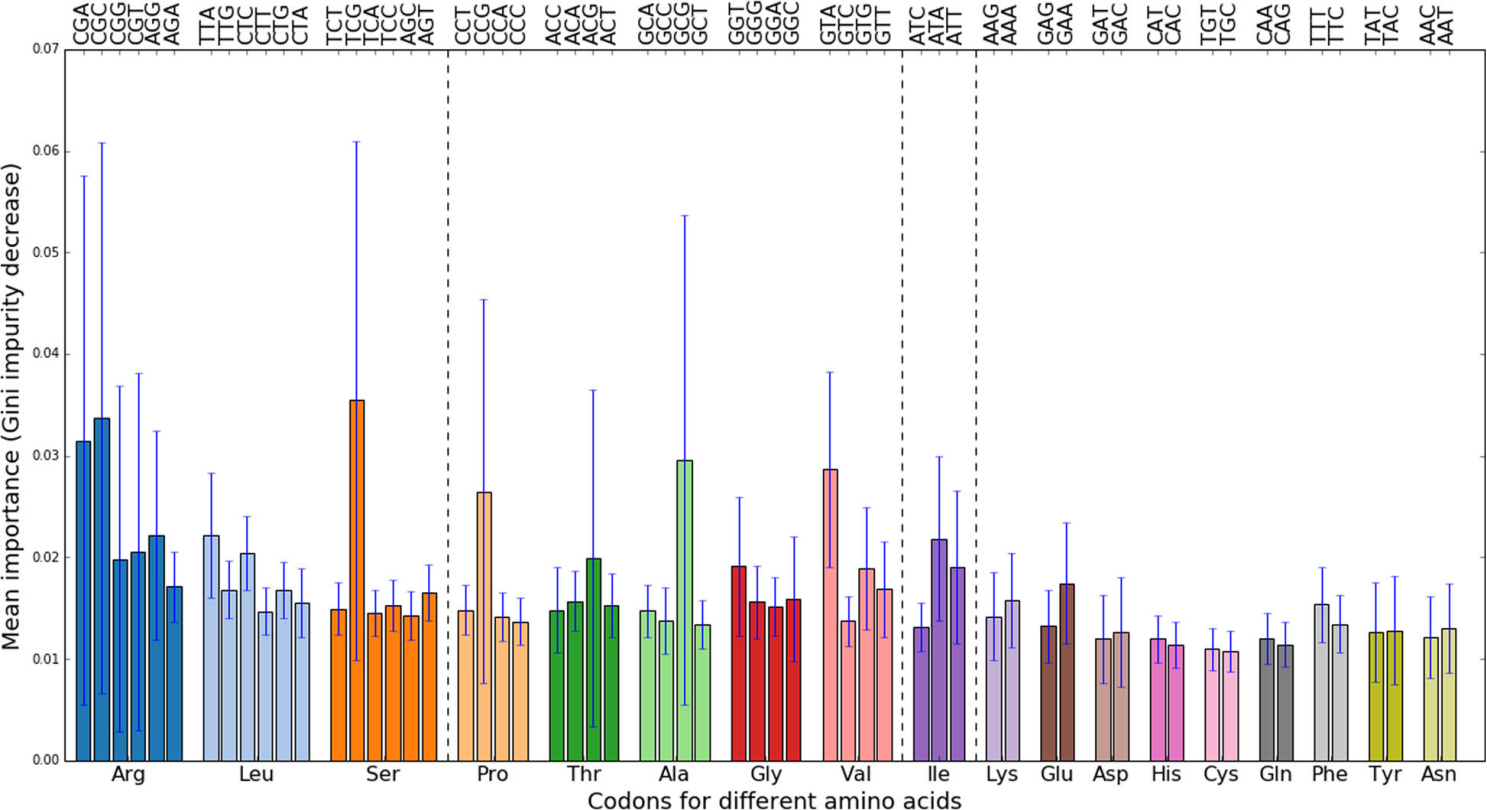
Feature importance in the Random Forest model as measured by Gini impurity decrease. Each RSCU value’s importance is averaged over a 1000 trees trained on the full metagenomics data set. Codons are grouped according to their corresponding amino acid, with the amino acids with most codons on the left. Vertical lines separate a.a.-s with 6 codons, 4, 3 and 2 codons. The variance is high due to correlations among synonymous codons. Codons TCG, CGC, CGA,GCG,GTA and CCG stand out as more important than others

The mean importance of each codon’s RSCU value for the virus detection task is displayed in Fig. 6.

The high variance in these mean importance values is driven by the correlations between the RSCU values of synonymous codons (see the explanation in “Methods” section). Despite the variance, it is clear that the sum importance of an amino acid seems to grow with increased number of triplets coding for it. Secondly, it can be seen that in most cases the importance is distributed rather uniformly across synonymous codons, but codons TCG (Ser), CGC (Arg), CGA (Arg), GCG (Ala), GTA(Val) and CCG(Pro) stand out as the most informative codons. As the classification algorithm treats all features as equals, such increased importance of certain codons might hint at underlying biological causes.

### Neural networks

To confirm and potentially improve the results from the RF classifier, we also trained a neural network (NN) classifier on the same RSCU data. We applied the same leave-one-experiment-out cross-validation technique, meaning 19 models were trained, each time leaving out data from one experiment. Table 2 summarizes the results for the best-performing (according to ROC area) neural network hyperparameters at classification threshold 0.5 using the same measures as for random forests. The results at threshold 0.5 for NN approach look superior to the results of random forests when comparing F1 scores for the viral class. However, area under the ROC curve reveals that the two methods are of equivalent power. The micro-averaged area under the ROC curve for this best model is 0.790, whereas the best random forest configuration reaches 0.789. Indeed compared to the random forests in Table 1 the neural networks in Table 2 seemed to trade off precision at the expense or recall, with the actual underlying power to discriminate (as measured by ROC area) staying the same.

**Table 2:** Micro- and macro-average performance measures across 19 experiments using feed-forward neural networks

Method	Class	Precision	Recall	F1-score
Micro-average	Non virus	0.97	1.00	0.99
Micro-average	Virus	0.69	0.13	0.21
Macro-average	Non virus	0.96	1.00	0.98
Macro-average	Virus	0.43	0.12	0.19

The results are from the best model according to area under the ROC curve (*ROC*_*micro*_=0.790). This model used two 1024 units FC layers with Relu nonlinearity, 0.25 dropout rate and *class*_*weight*_*power* 0.25 (see Additional file 3). All networks were trained for 10 epochs, using Adam optimizer with 10*e*−4 initial learning rate that was multiplied with 0.95 after each epoch

Thus both random forest and neural network models are able to detect patterns in the RCSU values that are predictive of the virus or non-virus nature of the samples.

## Discussion

We found that machine learning using RSCU can predict presence of viral contigs in metagenomic sequencing data.

Firstly, we investigated the possibility to use RSCU values to predict viral origin of sequences originating from complete protein coding genes at NCBI Genbank (the data was obtained from http://www.kazusa.or.jp/codon/). Using 10-fold cross-validation on this dataset, random forest models achieved 0.996 area under ROC curve on validation data. However, the models trained on this dataset failed to generalize when tested on metagenomics data - yielding only 0.510 area under ROC. Additionally, we tested the metagenomics model on the Genbank dataset, but results were also close to random guess. This failure probably occurs because metagenomic dataset is very noisy compared to the clean data obtained from Genbank. Instead of complete genes, it contains shorter fragments, it includes non-coding ORFs and has many sources of possible errors in the pipeline. Considering the differences between the two datasets, it is a logical outcome that a model built on one dataset does not perform on the other. As our goal is to detect viral sequences specifically in metagenomics data (and not just generally demonstrate predictability using RSCU values), we concluded that we should also train the model on metagenomics data.

Using both Feed Forward Neural Network and Random Forest classification methods and metagenomics as training dataset, we show that RSCU can predict the viral nature of a sequence in metagenomic dataset. While the method rediscovers only a small proportion of the viral contigs we nevertheless consider this a significant result because this rediscovery was achieved based solely on RSCU values extracted from ORFs (most of ORFs are probably not actually genes) without any additional external knowledge - such as a sequence database. Having used such very high-level features this method has a chance of generalizing outside the space of “known sequences” that it was trained on. This means that the presented solution can be applied to sequences that other, more informed methods leave unclassified because they are not similar enough to the “known sequences” in the database. This information is beyond the information offered by other methods and it significantly narrows down the search space for the discovery of unknown viruses in metagenomic samples.

We also investigated which codons played a decisive role, employing the Random Forest feature importance analysis. RSCU values for six codons (TCG (Ser), CGC (Arg), CGA (Arg), GCG (Ala), GTA(Val) and CCG(Pro)) were the most influential in the classification model. In the human genome, none of these 6 codons are frequently used [39]. In our metagenomics datasets, the average RSCU values for the top two influential codons, TCG and CGC, in non-viral contigs were also quite low (0.39 and 0.53), while in viral contigs they were more abundant (0.60 and 0.80). A similar pattern is also followed by the other four most influential codons (see Additional file 6 for a figure depicting mean RSCU values in the two classes). This indicates that the most decisive codons for the algorithm were the ones, which were least commonly found in non-viral sequences. It also suggests that the frequency of usage of these particular codons is different in viral and non-viral genome, which in turn hints at different biological characteristics of viral sequences. Further research will be necessary to analyze this difference.

Metagenomics datasets generated by NGS technologies from human biospecimens are noisy and contain many potential errors. After human samples are sequenced, the Illumina machine provides a vast amount of fragmented ’reads’ of DNA [40]. In order to reconstruct full genomes, de novo assembly algorithms are used, which introduces several types of errors, such as substitutions, insertions or deletions. These errors cause frame shifts in the potential coding regions that may greatly affect accuracy of RSCU values. Despite this highly noisy data (that comprised only 3% of viral sequences) our approach achieved 0.79 area under the ROC curve. In our bioinformatics pipeline, for the sequences that are classified as unknown by NCBI BLAST, the HMMER3 algorithm is applied. However, a large amount of sequences is still labeled as unknown where potential viral sequences might be hidden. Therefore, we propose that the machine learning models proposed in this work could be used as the third stage after BLAST and HMMER3.

De novo assembly for viral metagenomics is in its infancy and further improvements will most probably further enhance the predictive value of machine learning analysis of RCSU values for taxonomicclassification.

## Conclusions

The results of the present investigation indicate that simple counting statistics at the codon level and applying machine learning to RCSU values can provide important information in addition to conventional methods for taxonomic classification of sequences in metagenomic datasets. Future investigations should focus on a more flexible approach without pre-defined features (like RSCU values), such as training 1D convolutional neural networks on raw DNA sequence strings. This approach may have the potential to discover novel predictive features beyond codon usage and thus further improve the classification accuracy.

## Additional files

Additional file 1 Metadata of the samples used for this study. (XLSX 40 kb)

Additional file 2 Number of the assembled contigs classified into different taxonomy groups by BLAST. (XLSX 32 kb)

Additional file 3 Methods description in detail. (DOCX 19 kb)

Additional file 4 Results with different hyperparameters for random forest. (XLSX 13 kb)

Additional file 5 Results per experiment (random forest). (XLSX 32 kb)

Additional file 6 Figure summarizing mean RSCU values in the two classes. (PDF 64 kb)

Additional file 7 Counted RSCU values from metagenomic dataset. (TXT 59,594 kb)

## Abbreviations

FFN: Feed-forward neural network
LOEO: Leave-one-experiment-out cross validation
NGS: Next generation sequencing
NN: Neural networks
ORFs: Open reading frames
RF: Random forest
ROC: Receiver operating characteristic curve
RSCU: Relative synonymous codon usage
