# Supplementary material for: Machine Learning for detection of viral sequences in human metagenomic datasets
Source: BMC Bioinformatics. 2018 Sep 24;19:336. doi: 10.1186/s12859-018-2340-x (PMC6154907; doi:10.1186/s12859-018-2340-x)
Supplement: Supplementary file 3 — Methods description in detail. (DOCX 19 kb) [file 12859_2018_2340_MOESM3_ESM.docx]

**Machine learning for detection of viral sequences in human metagenomic datasets**

Zurab Bzhalava^1*^, Ardi Tampuu^2*^, Raul Vicente^2^  and Joakim Dillner^1^

^1^ Dept. of Laboratory Medicine, Karolinska Institutet, Stockholm, Sweden

^2^ Dept. of Computer Science, University of Tartu, Tartu, Estonia

* These authors contributed equally to this work.

Correspondence**:** Joakim Dillner, Dept. of Laboratory Medicine, Karolinska Institutet, F46, Karolinska University Hospital Huddinge, 141 86, Stockholm, Sweden. e-mail: joakim.dillner@ki.se

Machine Learning methods description in detail

Random forests

Random Forest [1] is a collection of a large number of decision trees, that differ from each other due to two sources of randomness added to the training process. First of all, each tree is not built using the training set of N samples as it is, but instead on N samples drawn with replacement from this set. For each tree some samples are used multiple times and some are disregarded. The other source of variability in a random forest is that at each potential splitting point, only a random subset of the features are considered. For classification problems it is common to use *sqrt(F)* features at a time, where F is the total number of features.

These two limitations make the trees more different from each other and enhance the forest’s ability to generalize as an ensemble. In the Python 2.7 scikit-learn-0.18.1 implementation[2] used in this work, the random forest classifier reaches a decision by averaging over the output probabilities of all the trees (in contrast to majority voting over trees as in original work by Breiman[1])

In our work we used the RandomForestClassifier from scikit-learn’s ensemble package with default parameters[2], except for:

- Using increased number of trees ([100,200,500,1000,5000]) instead of the default 10 trees. Using forests with 5000 trees, training 18 models using leave-one-experiment out cross-validation paradigm took more than 12 hours on 4CPUs (i5, 3.5GHz).
- In some runs using the parameter *class_weight= “balanced” ,* which automatically adjusts weights to be inversely proportional to class frequencies in the input data.

We also tested the effect of downsampling the majority class and upsampling the minority class. The results with different combinations of the aforementioned parameters (forest size, balancing of classes, sampling) are summarized in Additional File 4. While not reporting the results here, we confirm that we scanned the values also for some of the other influential parameters - *the depth of the trees*, *the number of features considered at each split* and *the minimum number of samples per leaf* - but none of the configurations we tested yielded significantly better results than the default values.

Artificial Neural Networks

Artificial Neural Networks is a machine learning algorithm inspired by the structure of the biological networks of neurons in the brain. The simplest type of artificial neural networks,  feedforward neural network[3], used in this work, consists of multiple layers of nodes (called “neurons”) and the connections between these nodes. There are no connections between the nodes of the same layer, whereas neighbouring layers are all-to-all connected with each other. Each node is characterized by its activation and each connection by its weight. The activations of the input layer nodes are set to be the values of the features of a data point. In our case the input features are RSCU values of a sequence. The activation values of the nodes in all subsequent layers are calculated by summing up the weighted inputs to that node and passing this sum through an activation function (non-linear function such as sigmoid, hyperbolic tangent and ReLu). The last layer, called output layer, contains as many nodes as there are outputs. In our case, it consists of 2 nodes that take values between 0 and 1 and are interpreted as the probabilities that the inputted RSCU values originate from a viral or a non-viral sequence.

By providing such network with pairs of RSCU features and corresponding virus/nov-virus labels, we can optimize the connection weights so that the outputs would match the true labels better and better. To do so, the gradient backpropagation algorithm[4] allows to find the derivatives of the prediction error with respect to the weights. Optimization methods then use these derivatives to change the weights little by little towards more useful values.

A random search, results not reported here, was performed to find the best hyperparameters for this task. We tested different values for [*number_of_hidden_layers, number_of_nodes, activation_function, optimization_algorithm,* *batch_size, number_of_epochs_trained, dropout_rate, learning_rate, learning_rate_decay_per_epoch and class_weight_power]*. All of the parameters, except the last on, are well known and influential hyperparameters. The *class_weight_power* parameter was introduced by us to better control how strongly we fight against class imbalance. In particular, the loss from samples belonging to the minority class is multiplied with:

(count_of_majority_class / count_of_minority_class) ^ class_weight_power ,

with counts from training set. Notice that if this power is set to 0, no class weights are applied. If this power is set to 1, we have balanced class weights, meaning the sum of weights for the two classes is the same. All real numbered values can be used, but in the random search for best parameters we used values from 0.0 to 2.0.

The results presented in the Results section are obtained with a network with two hidden layers of 1024 nodes each, dropout rate 0.25, learning rate 0.001, learning rate decay 0.95 per epoch, ReLu activation function and trained using Adam optimizer[5] for 10 epochs with batch size 100. Class weight power is set to 0.25 (also 0.0, meaning no class weights, gave comparable results).

The neural networks were implemented in Python 2.7.10 , using version 2.0.5 of Keras neural networks package[6] .

Averaging the results

Leave-one-experiment-out cross-validation approach leaves us with 18 different models and 18 validation sets of different size and prevalence. When making generalizations about our method’s ability to classify novel samples, we need to choose how to combine the results of different folds[7, 8]. Simply averaging the statistics achieved on different folds and disregarding the number of samples in each validation set is called macro-averaging[8]. This average across data sets tells us what performance to expect on RSCU data from a new unseen experiment. The option most often used in cross-validation is to aggregate the validation sets and calculate the metrics on this combined set. This approach is called micro-averaging[8] where experiments that provide more validation samples play more important roles. Low performance on datasets with few samples might go unnoticed. This average performance across samples gives us an idea of expected overall performance over many data sets from many different experiments.

Another factor to consider when analyzing the results is class imbalance. Across the 18 datasets, we have on average 3% of virus and 97% of non-virus samples. A naive model that classifies everything as non-virus would have a 97% overall precision and 97% overall recall. Despite rather high overall precision and recall, this model would nevertheless be useless for separating the classes. With high class imbalance we need to investigate precision and recall for both classes separately, instead of overall performance. This way we gain more insight to the model’s actual ability to detect viral samples.

The main metric for assessing the quality of classifiers in this work is however the area under the ROC curve. The area under Receiver Operating Characteristic (ROC) is a metric that does not depend on class distribution and is thus particularly useful for our highly unbalanced dataset.

Feature importance analysis

Each decision tree in a random forest is a collection of simple splitting rules (if-then statements) that use only one feature at a time. Another limitation is that at each splitting point only a small subset of features are considered. Among the possible one-feature if-then statements, the rule that maximally reduces Gini impurity is always chosen. Gini impurity is reduced if the two nodes resulting from the rule have less uniform class distribution than the parent node. How important a feature was in an entire tree can be estimated by summing up the impurity reductions brought about by this feature at all different branching points where it was used[1, 9]. The importance of each feature in each individual tree is calculated and easily accessible to the user in scikit-learn’s RandomForestClassifier^27^. To compare the importance of RSCU values of different codons for our classification task, we average the importance of the features across 1000 trees trained on the entire data set of all 18 experiments.

When interpreting the mean importances of features, we need to notice that the RSCU values of synonymous codons can be highly correlated - if value for one synonymous codon is high the other(s) must be low. If there are only two synonyms, the correlation is almost perfect. Correlated features “compete” for importance - the RSCU value that is used first in a given tree will have the chance to contribute the information shared between the correlated features and is likely to show up as more important[9, 10]. In a different tree the randomness might lead to another feature being selected first and contributing highly. This leads to high variance of feature importances across trees. Despite high variance, we believe the average importance is still interpretable and reveals which codons’ RSCU values are useful more often than others[9], especially when the differences are clearly visible.

**References**

1. Breiman L: **Random Forests**. *Machine Learning* 2001, **45**.

2. Pedregosa F, Ga, #235, Varoquaux l, Gramfort A, Michel V, Thirion B, Grisel O, Blondel M, Prettenhofer P *et al*: **Scikit-learn: Machine Learning in Python**. *J Mach Learn Res* 2011, **12**:2825-2830.

3. Bishop CM: **Neural Networks for Pattern Recognition**: Oxford University Press, Inc.; 1995.

4. Rumelhart DE, Hinton GE, Williams RJ: **Learning representations by back-propagating errors**. In: *Neurocomputing: foundations of research.* Edited by James AA, Edward R: MIT Press; 1988: 696-699.

5. Kingma DP, Ba J: **Adam: A Method for Stochastic Optimization**. *CoRR* 2014, **abs/1412.6980**.

6. Chollet F: **Keras**. 2015.

7. Forman G, Scholz M: **Apples-to-apples in cross-validation studies: pitfalls in classifier performance measurement**. *SIGKDD Explor Newsl* 2010, **12**(1):49-57.

8. Van Asch V: **Macro- and micro-averaged evaluation**

**measures**. 2013.

9. Strobl C, Boulesteix A-L, Kneib T, Augustin T, Zeileis A: **Conditional variable importance for random forests**. *BMC Bioinformatics* 2008, **9**(1):307.

10. Archer KJ, Kimes RV: **Empirical characterization of random forest variable importance measures**. *Computational Statistics & Data Analysis* 2008, **52**.
