# Supplementary material for: Machine Learning for detection of viral sequences in human metagenomic datasets
Source: BMC Bioinformatics. 2018 Sep 24;19:336. doi: 10.1186/s12859-018-2340-x (PMC6154907; doi:10.1186/s12859-018-2340-x)
Supplement: Supplementary file 6 — Figure summarizing mean RSCU values in the two classes. (PDF 64 kb) [file 12859_2018_2340_MOESM6_ESM.pdf]

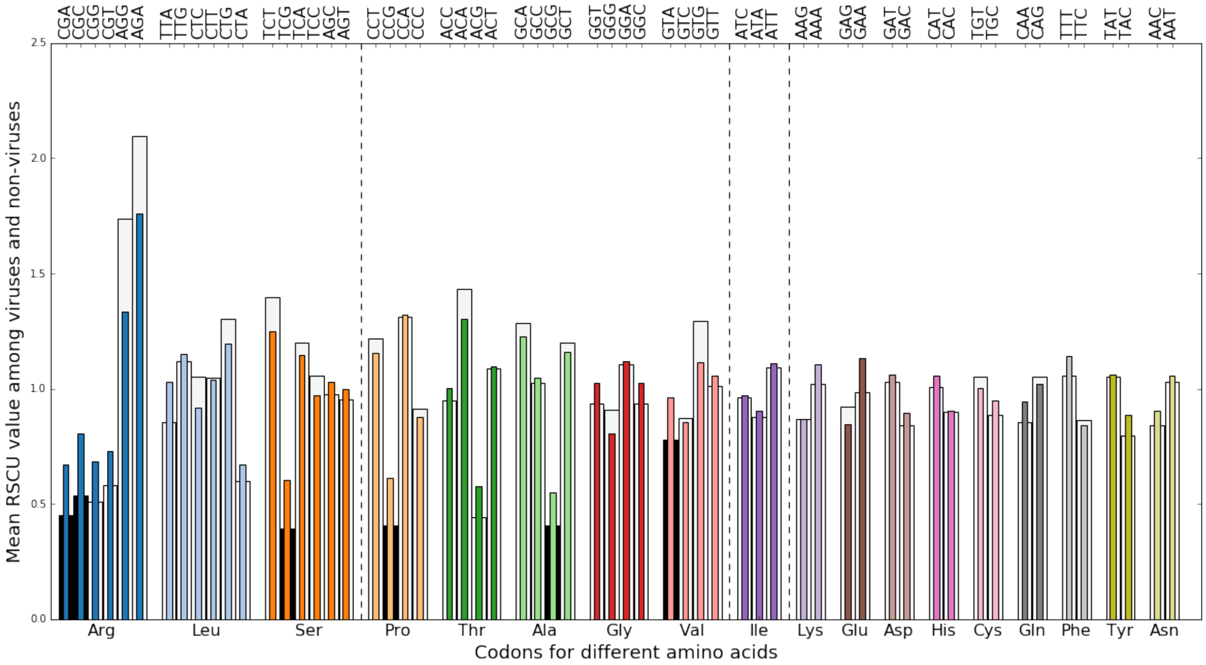

Figure 1: **Additional Figure: Mean RSCU values for virus and non-virus classes.** The mean values for each codon's RSCU value among the samples belonging to the virus class are depicted with narrow colored bars in the foreground. The mean values of each RSCU value among the non-viral data points are depicted with the wider bars in the background. The six bars colored black indicate the features (the RSCU values) that were the most important features according to the random forest feature importance.
